# Superb microvascular imaging (SMI) for evaluating hand joint lesions in patients with rheumatoid arthritis in clinical remission

**DOI:** 10.1007/s00296-018-4112-3

**Published:** 2018-07-30

**Authors:** Xiaolong Yu, Zun Li, Min Ren, Jing Xi, Jiabiao Wu, Yaxiang Ji

**Affiliations:** 1 Department of Ultrasound, Changzhou Wujin People’s Hospital, Affiliated with Jiangsu University, No. 2 of Yongning North Road, Changzhou City, Jiangsu Province China; 2 Department of Rheumatology, Changzhou Wujin People’s Hospital, Affiliated with Jiangsu University, Changzhou City, China

**Keywords:** Superb microvascular imaging, Rheumatoid arthritis, Power Doppler imaging, Remission

## Abstract

The utility of superb microvascular imaging (SMI) for evaluating hand joint lesions in patients with rheumatoid arthritis (RA) in clinical remission is unreported. This study aimed to compare SMI and power Doppler imaging (PDI) for the evaluation of hand joint lesions in these patients. Twenty-six patients with RA in clinical remission were enrolled. A total of 572 joints (52 wrist, 260 proximal interphalangeal, and 260 metacarpophalangeal joints) were detected by SMI and PDI. A semi-quantitative scale of 0–3 was used to compare the detection of synovial blood flow signal by SMI and PDI. Inter-observer agreement for the assessment of SMI and PDI scores was measured with kappa values. In the ten healthy volunteers, SMI and PDI signals were both scored 0. In the 26 RA patients, the remission rate via PDI was 65.4% but was only 42.3% via SMI. SMI also detected microvessel flow signal in seven patients diagnosed with clinical remission via PDI. Moreover, a total of 106 blood flow signals (18.5%) were detected by SMI, while 50 blood flow signals (8.7%) were detected by PDI. Compared with PDI, SMI increased 18.0% of power flow signals from Grade 0–1 and increased 13.7% of power flow signals from Grade 1–2. One joint classified as Grade 1 by PDI was classified as Grade 0 by SMI. Inter-observer agreement for PDI and SMI semi-quantitative scoring was moderate (kappa = 0.463). SMI seems more sensitive than PDI for detecting hand joint lesions in RA in clinical remission PDI, and could aid the achievement of true remission in RA patients.

## Introduction

Rheumatoid arthritis (RA) is an autoimmune disorder characterized by polyarticular inflammation and pannus formation, leading to joint destruction and severe disability [1, 2]. Remission or low disease activity is the ultimate aim for the treatment of RA [3, 4]. Moreover, in the past few years, several remission criteria have been established including clinical and biological criteria [5]. However, several studies have shown infra-clinical synovitis and disease exacerbation has persisted in patients who achieved clinical remission [6]. Thus, it is important to identify true remission in RA patients.

Imaging modalities, such as magnetic resonance imaging, have been reported to detect persistent inflammation in RA patients in clinical remission; however, they require contrast enhancement [7, 8]. In addition, power Doppler imaging (PDI) can detect modifications in synovial vascularity but it is not very sensitive to microvascular patterns and low blood flow velocity [9]. Superb microvascular imaging (SMI) is a recent innovative and effective ultrasound (US) Doppler modality, which can visualize low-velocity flow in microvessels. SMI uses a new adaptive algorithm to extract flow signals from large to small vessels and has been used in the diagnosis of breast, thyroid, and urinary tract infection [10–13]. Moreover, SMI can detect synovial inflammation in rheumatic diseases [14]. However, to date, the utility of SMI for evaluating hand joint lesions in patients with RA in clinical remission has not been reported.

The present study aimed to investigate SMI signals in the hand joint of patients with RA in clinical remission and compare the findings with those of PDI. We also aimed to demonstrate the value of SMI for identifying true remission in RA.

## Method

### Patients

Twenty-six consecutive outpatients and inpatients with RA in clinical remission were recruited for this study from January 2016–December 2017. All patients fulfilled the 2011 American College of Rheumatology/European League Against Rheumatism (ACR/EULAR) diagnosis criteria for RA, and two definitions for the clinical remission criteria were proposed as follows: (1) swelling joint counts, tender joint counts, C-reactive protein (mg/L), and patient global self-assessment (all four ≤ 1); and (2) simplified disease activity index (SDAI) ≤ 3 [15]. Exclusion criteria were: (1) congenital abnormalities or a history of trauma in the hand joints; (2) joint swelling pain caused by connective tissue diseases and other unknown reason.

Ten healthy volunteers (220 joints) without any history of RA as controls participated in this study. Exclusion criteria were: (1) congenital abnormalities or a history of trauma in the hand joints; (2) synovial hyperplasia in the hand joints.

### US examination

The wrist, proximal interphalangeal, and metacarpophalangeal joints of both hands (total 572 joints: 52 wrist, 260 proximal interphalangeal, and 260 metacarpophalangeal joints) were assessed in the axial and longitudinal planes using a 5–14 MHz broadband linear transducer (Aplio 500 US system, Toshiba Medical Imaging, Japan). The distribution and thickness of the synovium were observed by two-dimensional ultrasound, the position of the probe was fixed with a thick section of the synovial membrane, then we activated the power Doppler mode, and the blood flow scale and blood flow sampling frame were adjusted to appropriate, maximized the color gain but there is no false color displayed, the image is stored at the same time. Switch to SMI mode, we kept parameters such as 2D gain, color gain, blood flow scale, sampling frame size, and dynamic range unchanging, then recorded the distribution of SMI synovial blood flow on the same plane and stored the image. All examinations and assessments were performed by one rheumatologist who had more than 3 years of experience in US diagnosis and blinded to the clinical information and laboratory data. The synovial blood flow signal detected by SMI and PDI were scored on a semi-quantitative scale of 0 to 3: Grade 0 (no blood flow signal in the synovial membrane); Grade 1 (1–2 blood flow signal); Grade 2 (3–4 short linear blood flow signal in less than one-half of the synovial membrane); and Grade 3 (blood flow signal in more than one-half of the synovial membrane) [16].

### Statistical analysis

Data in this study were analyzed with SPSS 17.0 (IBM, Armonk, NY, USA). The chi-squared test was used to assess the difference between SMI and PDI, and a *p* value < 0.05 was considered statistically significant. Data are presented as the mean ± standard deviation or median value and interquartile range (25th–75th percentiles). Inter-observer agreement for the assessment of SMI and PDI scores was measured with kappa values. Interpretation of kappa was as follows: kappa < 0: less than chance agreement; kappa 0.01–0.20: slight agreement; kappa 0.21–0.40: fair agreement; kappa 0.41–0.60: moderate agreement, kappa 0.61–0.80: substantial agreement; and kappa 0.81–0.99: almost perfect agreement.

## Results

### Demographic characteristics

The clinical characteristics of the RA patients in remission and healthy volunteers involved in the study were showed in Table 1. There were no statistically signifcant differences in age, gender, RF, CRP and ESR between the two groups. In addtion, in the ten healthy volunteers, SMI and PDI signals were both scored 0 in the 220 joints, and no trauma, arthredema, synovial hyperplasia or synovitis was observed.

**Table 1:** Demographic characteristics of RA and HV in this study

Clinical data	RA	HV	*P* value
Number	26	10	
Age, years^a^	40.5 ± 9.2 (33–68)	41.2 ± 10.3 (32–71)	0.833
Gender (F/M)	24/2	9/1	0.347
Disease duration (years)^b^	17.2 (3–30)	None	
Morning stiff time (min)^b^	3 (0–10)	None	
RF (IU/ml)^b^	10.6 (4.7–13.2)	7.5 (2.0–9.6)	0.113
CRP (mg/L)^b^	1.85 (0.32–20.5)	2.12 (0.49–8.9)	0.224
ESR (mm/h)^b^	10.0 (3–29)	8.9 (4–15)	0.092
SDAI^b^	1.3 (0.1–3.0)	None	
Treatment with biological agents (%)	17 (65.4)	None	

*RA* rheumatoid arthritis, *HV* healthy volunteers, *CRP* C-reactive protein, *ESR* erythrocyte sedimentation rate, *SDAI* simplified disease activity index

^a^Mean ± SD (range)

^b^Median (range)

### Comparison of the remission rate in PDI and SMI

Table 2 compares the remission rate as evaluated by SMI and PDI in the 26 patients. PDI showed 9 patients (34.6%) with synovial inflammation and 17 patients in remission (remission rate 65.4%). However, SMI showed 15 patients with synovial inflammation and 11 patients in remission (remission rate 42.3%). Moreover, SMI detected microvessel flow signal in seven patients diagnosed with clinical remission by PDI. According to these results, SMI revealed that patients with RA in clinical remission did not achieve real remission. The difference in the detection of remission rate between SMI and PDI was statistically significant (*χ*^2^ = 5.488, *P* = 0.019).

**Table 2:** Comparison of the remission rate in PDI and SMI

SMI	PDI		Total	*χ* ^2^	*P*
	+	**−**			
+	8	7	15	5.488	0.019
**−**	1	10	11		
Total	9	17	26		

*SMI* superb microvascular imaging, *PDI* power Doppler imaging

+ positive result, − negative result

### Comparison of the presence of synovial SMI and PDI signals

SMI and PDI further detected the synovial blood flow signal in the 572 hand joints of the 26 patients. Table 3 shows that SMI detected 106 blood flow signals (18.5%), while PDI detected 50 blood flow signals (8.7%). SMI, compared to PDI, revealed the presence of synovial blood flow signal in a significantly greater number of joints in patients with RA in clinical remission (*χ*^2^ = 229.1, *P* < 0.001).

**Table 3:** Comparison of the presence of synovial SMI and PDI signals

cSMI	PD		Total	*χ* ^2^	*P*
	+	**−**			
+	49	57	106	229.1	< 0.001
**−**	1	465	466		
Total	50	522	572		

*SMI* superb microvascular imaging, *PDI* power Doppler imaging

+ positive result, − negative result

### Comparison of the grades by SMI and PDI

**Fig. 1:**
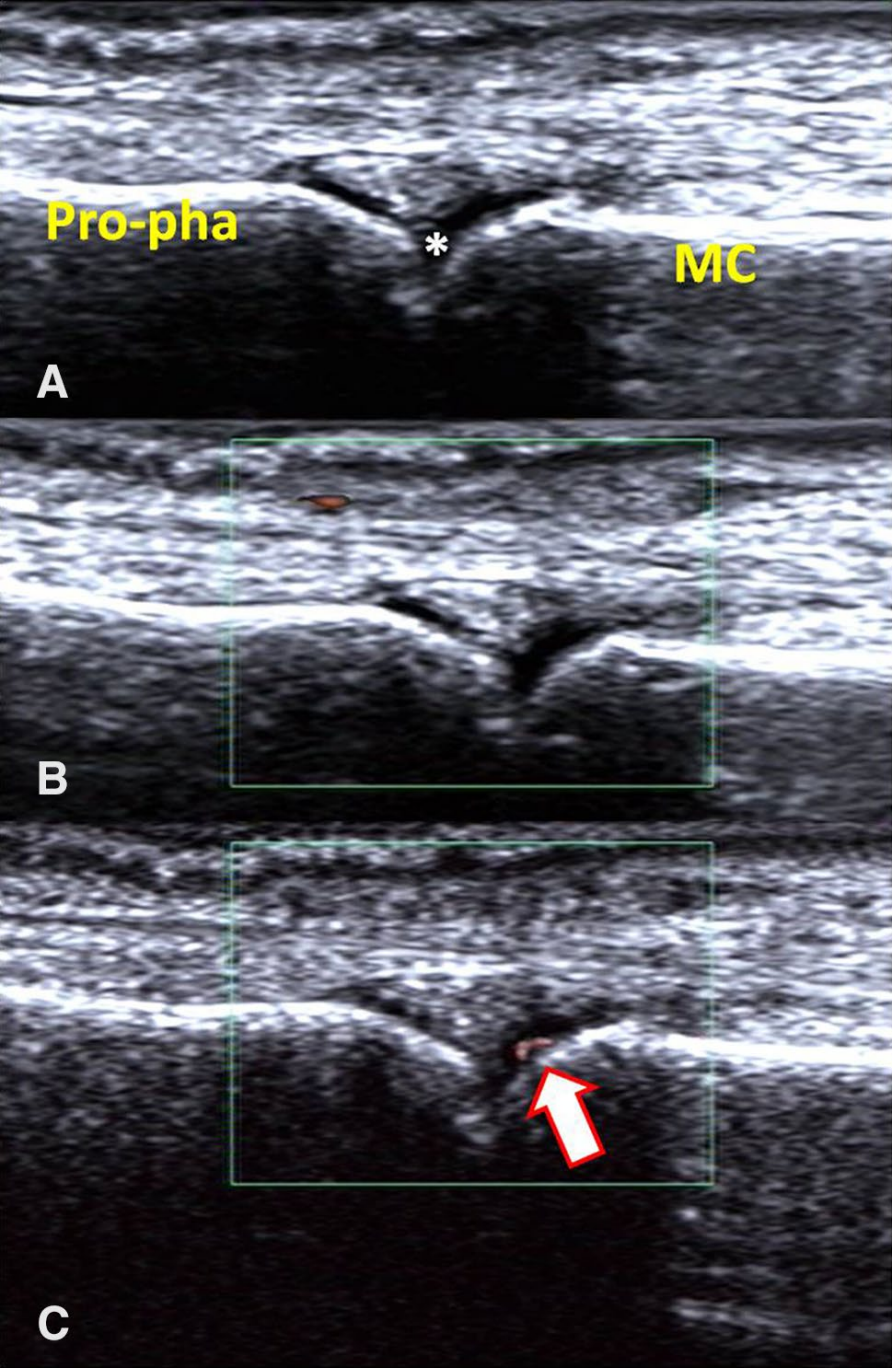
**a** synovial hyperplasia was clearly seen in the second metacarpophalangeal joint of right hand in a patient within clinical remission after RA treatment (*synovial hyperplasia; *MC* metacarpal, *Pro-pha* proximal phalanx). **b** No blood signal was seen in PDI. **c** Linear blood flow signal could be seen in the hyperplastic synovial membrane in SMI

Using the semi-quantitative scale of 0–3, Table 4 shows the comparison of the grades of synovial blood flow in the 572 hand joints via SMI and PDI. Regarding SMI, 18.0% of the power flow signals increased from Grade 0–1, while 13.7% increased from Grade 1–2. Moreover, one joint classified as Grade 1 via PDI was classified as Grade 0 via SMI. Inter-observer agreement for the assessment of PDI and SMI semi-quantitative scoring was moderate (Kappa = 0.463, *P* < 0.01). These results demonstrated that SMI was more sensitive than PDI for detecting synovial vessel signals of the hand joint in patients with RA in clinical remission. Figure 1 illustrates the case of a woman patient (45 years old) evaluated with SMI and PDI.

**Table 4:** Comparison of the grades by SMI and PDI

PDI	SMI				Total
	0	1	2	3	
0	402	90	7	0	499
1	1	62	10	0	73
2	0	0	0	0	0
3	0	0	0	0	0
Total	403	152	17	0	572

Difference between SMI and PDI is statistically significant (*P* < 0.01)

*SMI* superb microvascular imaging, *PDI* power Doppler imaging

## Discussion

RA, characterized by erosive synovitis, causes irreversible bone damage and loss of function. Thus, remission is important for RA patients and persistent subclinical synovitis in RA patients who achieve clinical remission highlights the importance of true remission [17]. In the present study, we showed that SMI, a new microvascular flow imaging modality, was more sensitive than PDI for detecting synovial vessel signals of the hand joint in RA patients who achieved clinical remission, suggesting that SMI has great potential for improving diagnostic accuracy in evaluating RA remission.

Although the 2011 ACR/EULAR remission criteria have been developed as guidelines for clinical remission, several studies have proposed the use of imaging remission [17, 18]. A recent study showed that in RA patients with clinical remission, power Doppler activity was demonstrated in the dominant hand and wrist in approximately half of the patients via US examination and the risk of recurrence was 4.5 greater for those with power Doppler positivity than for those with power Doppler negativity [19]. In addition, the recurrence rate of RA in patients who achieved imaging remission was significantly lower than that of patients who did not achieve imaging remission [20]. Our present results showed that in the 10 healthy volunteers, SMI and PDI signals were both scored 0, while in 26 RA patients with clinical remission, the imaging remission rate was 65.4% by PDI and only 42.3% by SMI, consistent with the previous study. Thus, imaging remission could be used to improve the prognosis of RA patients.

Our results further showed that SMI detected more synovial blood flow signal in the 572 hand joints compared with PDI, and improved the blood signal classification to some extent, suggesting that SMI could be more sensitive than PDI for detecting synovitis of the hand joint in RA patients with clinical remission. The moderate inter-observer agreement between PDI and SMI indicates that SMI is a feasible and reliable technique. In recent years, SMI has been reported to allow the visualization of low-velocity flow in microvessels excluding the use of contrast agents, high costs, and invasiveness [21]. Moreover, several studies have reported that SMI, compared to PDI, significantly improved the detection of blood flow signal and synovial inflammation within the joints in RA patients [13, 14]. And our present study is the first to evaluate SMI in RA patients with clinical remission. Compared with PDI, SMI significantly improved the detection of synovial blood flow signals.

In our study, a patient was classified as having Grade 1 synovial blood flow by PDI but was classified as Grade 0 by SMI. We found the patient was in the motor neuron obstacle treatment center in our hospital and had a history of RA. Thus, because of the interference of the tissue movement, PDI might have produced the pseudo-image. PDI is an important method for evaluating RA synovitis and for detecting vascularity in the joint of RA patients [22–24]. However, it is limited in the detection of microvascular patterns and low blood flow velocity [25]. Our results indicated that SMI does not have this PDI limitation and is of great value for identifying true remission in RA patients.

Our present study has several limitations. First, the sample of patients was relatively small. Second, we just only detected wrist, proximal interphalangeal, and metacarpophalangeal joints to asses hand joint lesions. Third, the hand joint synovitis alterations were graded only based on the PDI or SMI score, without contrasting with the pathology.

In conclusion, our results suggest that SMI is more sensitive than PDI for the detection of hand joint synovitis in RA patients who have achieved clinical remission, and could aid RA patients to achieve true remission. Further studies are needed to validate the role of SMI in improving diagnostic accuracy in RA remission.
